# Unraveling the causes of the Seoul Halloween crowd-crush disaster

**DOI:** 10.1371/journal.pone.0306764

**Published:** 2024-07-12

**Authors:** Haoyang Liang, Seunghyeon Lee, Jian Sun, S. C. WONG

**Affiliations:** 1 Key Laboratory of Road and Traffic Engineering, Ministry of Education, Tongji University, Shanghai, China; 2 Department of Civil Engineering, The University of Hong Kong, Hong Kong, SAR, China; 3 Department of Transportation Engineering, University of Seoul, Seoul, Korea; National University of Sciences and Technology NUST, PAKISTAN

## Abstract

As the world steadily recovers from the COVID-19 pandemic, managing large gatherings becomes a critical concern for ensuring crowd safety. The crowd-crush disaster in Seoul in 2022 highlights the need for effective predictive crowd management techniques. In this study, an empirical analysis of this incident is conducted using data from various sources, and model-based simulations are created to replicate hazardous crowd conditions in high-risk areas. In the empirical analysis, mobile device data indicates a significant increase in population above normal levels in the disaster area just hours before the incident occurred. In the simulations, a hydrodynamic model is employed to simulate a bidirectional collision, which quantitatively demonstrates that the average density during the crush reached **7.57 ped**/**m**^**2**^ (with a maximum of (**9.95**)**ped**/**m**^**2**^). Additionally, the average crowd pressure peaked at **1,063 N**/**m** (with a maximum of **1,961 N**/**m**), and the maximum velocity entropy was **10.99**. Based on these findings, it can be concluded that the primary causes of the disaster were the substantial population, bidirectional collision, and escalating panic. The results of controlled simulations under various management strategies are then presented. By implementing effective crowd management techniques, crowd safety can be enhanced through quantitative comparisons of these key indicators.

## Introduction

As the COVID-19 pandemic subsides, many countries have witnessed a resurgence in travel demand [1–3], accompanied by the return of major events and mass gatherings. Nonetheless, amidst this heightened activity, addressing the long-standing issue of crowd safety remains critical, considering the tragic history of numerous fatalities linked to such gatherings [4]. Historical crowd disasters have transpired in various scenarios, including concert venue situations like the 2010 Love Parade disaster, which caused 21 fatalities and 652 injuries during a music festival [5]. Another significant context for such tragedies is religious gatherings, as exemplified by the 2015 Hajj pilgrimage, which witnessed a devastating crowd disaster with reports indicating over 2,400 fatalities during the event [6]. The recent Seoul Halloween crowd-crush disaster [7], occurring in Seoul’s Itaewon neighborhood on October 29, 2022, during a post-pandemic Halloween celebration, led to a tragic loss of lives and injuries. This incident serves as a stark reminder of the inherent risks and challenges in managing crowd safety during similar events, emphasizing the urgent need for effective disaster preparedness and management strategies.

The Itaewon neighborhood, located in the Yongsan-gu district of Seoul, Korea, is a popular nightlife destination in the city. On October 29, 2022, an unusually large gathering took place in Itaewon. Long-term evolution (LTE) mobile device data [8] revealed that over 38, 000 visitors were trapped within the neighborhood. This figure significantly exceeds, by more than threefold, the average actual populations recorded in the area throughout October 2022. In addition to the risks posed by the swelling crowd, frequent police calls noted in the official report [7], indicated an escalating sense of panic in the critical region. Despite the population data and psychological alerts prompting the implementation of crowd management strategies, the disaster still occurred. This raises questions: Was the crowd disaster inevitable? Could it have been prevented? This study reexamines the Seoul Halloween crowd-crush by conducting a comprehensive empirical analysis and employing mechanism-informed crowd simulations. The objective is to uncover the key contributing factors and identify targeted crowd management strategies.

Over the years, empirical studies of crowd disasters have illuminated the quantitative relationships between critical crowd states in hazardous situations [5, 9, 10]. These relationships are invaluable for creating a comprehensive map of intricate movement patterns, identifying potential hazards, and simulating proposed strategies for crowd management. However, deciphering and predicting crowd behavior remains a formidable challenge due to the inherent complexities and uncertainties in crowd dynamics. Addressing this challenge requires a cohesive solution that combines data-based tools [11] with model-based evaluations [12]. On one hand, the multifaceted data collected during disasters, such as geometric information and social media usage [13], complicates the task of estimating crowd situations. Extracting meaningful information from this data and converting it into suitable inputs for prediction and evaluation is a significant need in the design and implementation of crowd management strategies. On the other hand, the primary complexity in modeling dense crowds stems from understanding the formation and impact of crowd pressure and turbulence. These characteristic flocking patterns in crowd dynamics, which directly trigger stampedes and related fatalities, are influenced by numerous physical and psychological factors [14, 15].

In situations of high crowd density, the concept of crowd pressure becomes particularly evident. The frequency of physical interactions between individuals significantly escalates, facilitating the propagation of force. This force can rapidly accumulate when it encounters resistance from surrounding obstacles, interaction forces among individuals, and friction between the crowd and the ground. This phenomenon is often referred to as the formation of “force chains” in crowds [16]. As expected, these pushing forces propagate in the anticipated direction of movement, generating exceptionally high pressure at the front of the dense crowd. Examples include the pole during the Love Parade Crowd disaster [5] and the collision area between bidirectional pedestrian streams such as the middle of the alley in the Seoul Halloween crowd-crush [17]. Previous research has indicated that sustained high pressure on individuals can result in fatalities, serious injuries, and significant psychological harm [18]. Post-disaster assessments have shown that during crowd disasters, the compressive forces exerted on individuals by crush barriers can reach 1000 N/m, causing severe discomfort and injuries [19]. In addition to the physical damage, crowd forces are also believed to inflict psychological harm. A recent questionnaire survey demonstrated that the force perceived significantly affects perceived safety [20], contributing to a more panicked crowd before the crowd crush occurs.

Crowd turbulence, another direct cause of stampedes, refers to the chaotic movement of pedestrians in a crowd. This phenomenon can be directly observed through time-lapse photography during crowd disasters, such as the 2006 Hajj stampede [15]. Shock waves resulting from crowd turbulence can be distinguished from the stop-and-go waves observed in normal pedestrian flow by the following features [16]: 1) consistently high crowd density, 2) random and unintended irregular motions, and 3) varying strength and direction of high crowd pressure. In typical congestion scenarios, as pedestrian density increases, the flow rate depicted in the pedestrian flow-density relationship decreases until the crowd comes to a standstill. However, in panicked cases, after the flow rate decreases for a while, it increases again, creating a “second peak” in the diagram [9, 16]. This second peak is usually associated with crowd turbulence, where pedestrians are forced to move more synchronously, often due to external pressures or constraints. This can trigger sudden, coordinated surges of movement, which escalate the potential risk of falls and stampedes.

In this study, data were initially collected from multiple sources to compile an empirical report detailing the progression of the disaster. Mobile device data played a crucial role in providing early warnings about the accumulation of large populations in confined areas, while police calls offered insight into the escalation of crowd panic. Incorporating a dynamic estimation of Origin-Destination (OD) flow assignment, these inputs served as significant factors for the proposed model-based prediction and evaluation framework to reproduce the Seoul Halloween crowd-crush, illustrating collision points, high density, extreme pressure, and crowd turbulence. Ultimately, a dangerous crowd condition was simulated, with a maximum density of 9.95 ped/m^2^, physical pressure of 1961 N/m, and a maximum velocity entropy (VE) of 10.99 in the critical region. Following this, a crowd management strategy was implemented within the post-disaster analysis framework, aiming to avert bidirectional collisions. The prediction and evaluation results underscored the effectiveness of this strategy in enhancing network capacity and mitigating crowd risk. Through these efforts, this study sheds light on the direct and underlying causes of the Seoul Halloween crowd-crush and presents practical management strategies to prevent such occurrences in the future.

## Investigation and results

### An empirical report of the disaster

The Itaewon neighborhood, located in the Yongsan-gu district of Seoul, Korea, is one of the city’s most popular nightlife destinations. It attracts both locals and tourists, offering a diverse range of entertainment options, such as bars, clubs, and restaurants. On October 29, 2022, Itaewon became the epicenter of Halloween celebrations, drawing people from Seoul and around the world to take part in the festivities. This event was particularly significant, given the recent lifting of COVID-19 pandemic restrictions in Korea, which allowed people to engage in social activities once again.

**Fig 1:**
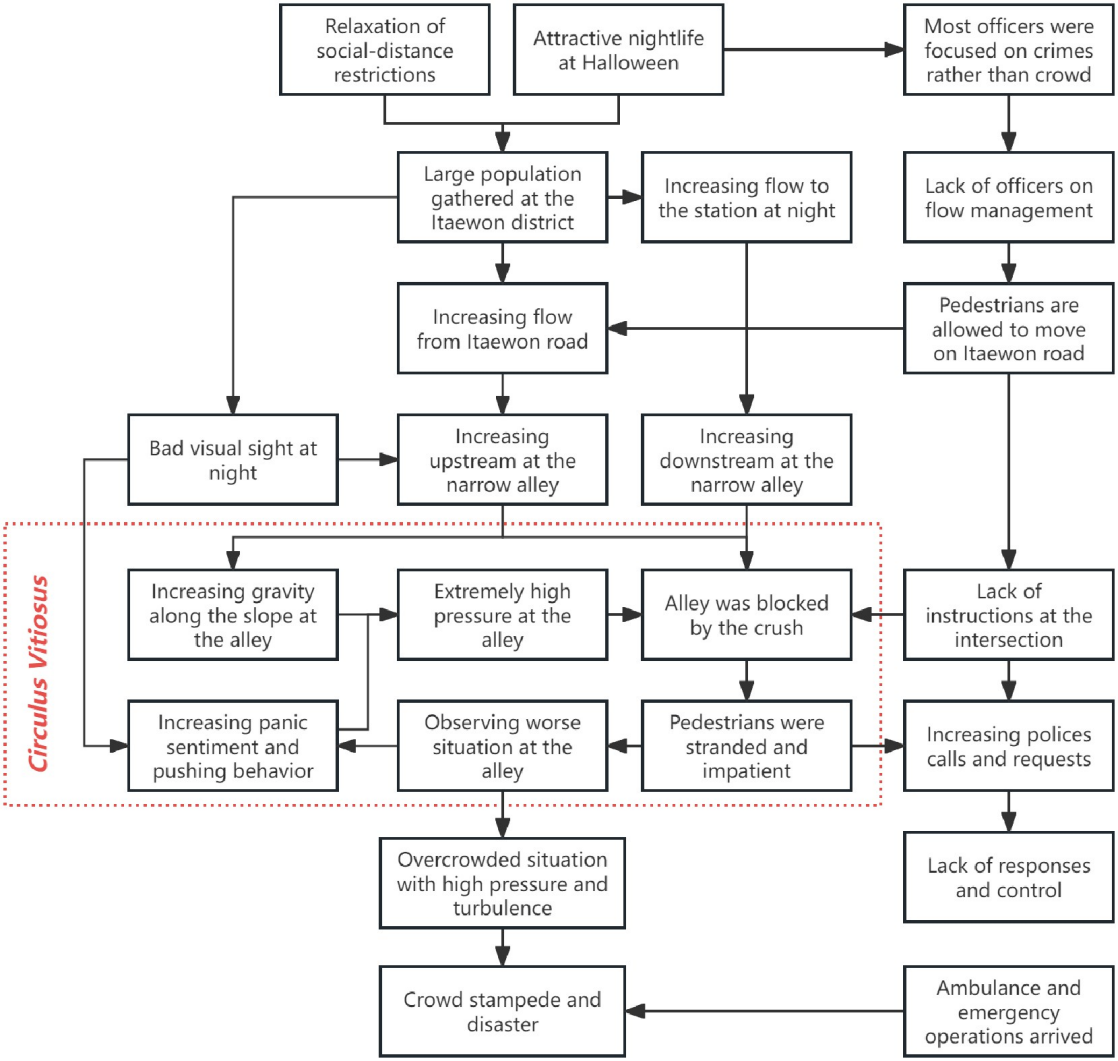
Illustration of the relevant causes and events before the crowd crush during the Seoul Halloween. The evolution of crowd dynamics was influenced by a multitude of endogenous and exogenous factors. Exogenous factors included elements such as flawed crowd management strategies, poor visibility at night, and hazardous geometry near the alley. Conversely, endogenous factors encompassed aspects such as large crowd size, heightened panic, and intense pressure.

**Fig 2:**
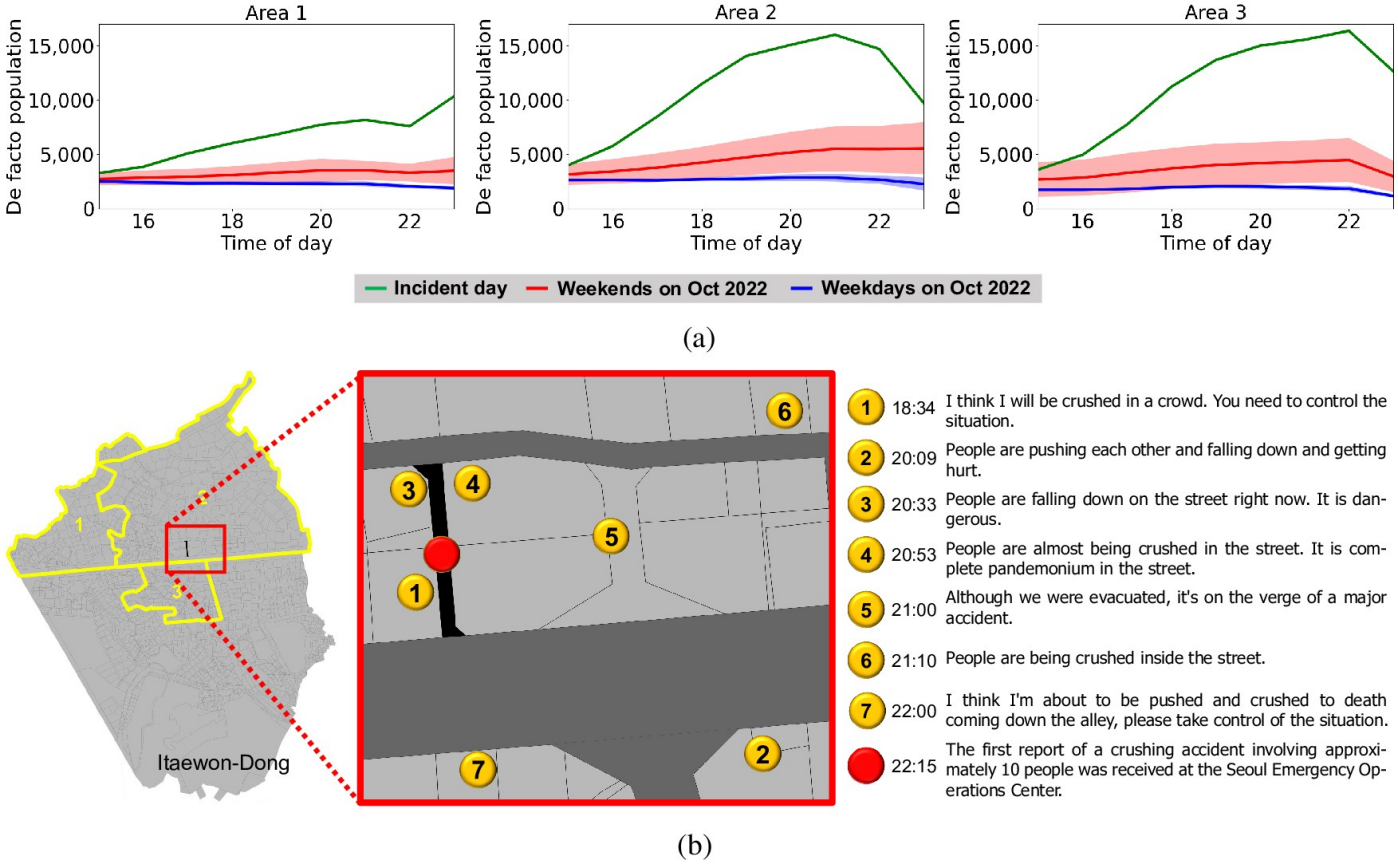
Evolution of the situation in the Itaewon neighborhood on October 29, 2022. (a) Evolution of the actual populations in the three areas. (b) A condensed timeline and locations of police calls received at Itaewon-Dong.

As depicted in Fig 1, this study outlines the process of the incident using available data. On that day in Seoul, a massive crowd congregated to partake in holiday celebrations. This event drew a considerable number of participants, leading to a situation of high crowd density. The situation was particularly dire in the alley (Fig 2) where pedestrians from Itaewon-ro (south street) and Itaewon-ro 27ga-gil (north street) met. This alley, with an average width of less than 4 meters, quickly became a bottleneck as the flow of people intensified. The narrow passageway, combined with the darkness of the night, hindered individual movement and visibility, thereby exacerbating the overall sense of panic among pedestrians. As the crowd grew increasingly panicked [21], behaviors such as screaming and pushing further intensified the chaos. The high density and pressure from individuals at the rear of the crowd, unaware of the front situation, also contributed to the disorder. This “Circulus Vitiosus”, reminiscent of the 2010 Love Parade crowd disaster referenced in [5], escalated into a crowd crush, resulting in numerous injuries and fatalities.

Fig 2(a) illustrates the population evolution in Itaewon, based on the analysis of long-term evolution (LTE) mobile device data collected at each base transceiver station by the Seoul Big Data center [8]. LTE data was gathered from three areas (Area 1, Area 2, and Area 3) in the Itaewon neighborhood, each exhibiting different population-evolution patterns. Normally, the population during weekends is slightly higher than on weekdays. However, on this particular day, the population surge in Itaewon was exceptional. The actual populations in these areas during the incident rose to levels significantly higher than their respective average populations for the month of October 2022. Specifically, the population in Area 2 increased by 380%, while Area 3 experienced a staggering increase of 650%. This dramatic surge in population density played a significant role in the unfortunate crowd-crush that ensued.

The lack of detailed planning for large crowd management during the Itaewon Halloween festival, as revealed in the final report [17], is another key contributor to the crowd crush. The agencies involved accurately anticipated a larger crowd due to the event falling on a weekend and the reopening of clubs and entertainment venues after a year of pandemic-induced closures. They also correctly expected an increase in emergency calls to the police. However, their response strategy was narrowly focused on strict enforcement against illegal activities and unruly behavior, overlooking specific details regarding large crowd response, support from police mobile units, and deployment strategies. Furthermore, certain crowd management decisions worsened the situation. From 18:30, the crowd was allowed to use the driveways on the south street, a decision intended to increase traffic capacity [22]. However, this strategy inadvertently intensified the flow from the south street to the narrow alley, thereby worsening the congestion in the alley. This highlights the importance of a comprehensive approach to crowd management that not only considers the immediate effects but also the broader impact on the surrounding areas.

The growing panic evident in police calls between 18:34 and 22:11 highlights the psychological factors contributing to the crowd crush [21]. As shown in Fig 2(b), a total of eleven overcrowding-related calls were received during this period. After the third call, fifty personnel were dispatched to various police stations, and additional officers were deployed to the exits of Itaewon Station following the sixth and eighth overcrowding calls. Besides, at 21:34, twenty military police officers were deployed to manage traffic. Despite these efforts, three more overcrowding calls were made, indicating that the situation remained unresolved. The incident occurred at 22:15, prompting the Yongsan Police Station chief to order all available personnel to the scene at 22:18. The following points highlight three critical moments during the disaster and suggest the rising panic within the crowd:

1. At 18:34, during the first overcrowding-related call to the police from the alley where the disaster occurred, a person reported that people were at risk of being crushed in the crowd and urged the police to take control of the situation. The caller stated that pedestrians were already congested in the lower half of the alley, and although the situation was still under control, pedestrians were beginning to feel impatient.
2. At 20:09, a person called the police to report that people were pushing each other, falling down, and getting hurt. Such pushing behavior emerged after prolonged congestion, indicating that panic sentiment had reached a high level. Furthermore, injuries resulting from falls suggested the presence of pressure and turbulence in the crowd.
3. At 22:15, the first report of a crushing accident involving approximately 10 people was received at the Seoul Emergency Operations Center. After the initial report, emergency calls continued until 22:28 when the first emergency rescue team arrived. The end of the crowd crush corresponded with the population’s evolution around 10 to 15 minutes after the accident (Fig 2(a)).

Utilizing data from mobile devices, government records, and overcrowding-related calls made before and during the disaster, a comprehensive timeline of events leading up to the incident has been constructed, as detailed in the S1 Table. This empirical report allows us to identify several human factors indirectly contributing to the crowd crush. Firstly, an unusually large crowd gathered in the Itaewon neighborhood due to the Halloween festival being held without physical distancing measures. Secondly, a flawed crowd management strategy, rerouted pedestrians from the sidewalk to the driveway markedly amplified the pedestrian flow along the south street. This strategy played a significant role in worsening congestion near the alley. Lastly, despite numerous calls to police emergency services that included reports of asphyxiation, requests for crowd control, and desperate pleas for help, the authorities failed to adequately recognize the escalating panic and respond appropriately. This indicates a significant lack of psychological relief measures.

### Model-based reproduction of key dangerous states

Considering that individuals’ intentions are unlikely to disrupt the fluid-like movement of a crowd when its density increases to over 7 ped/m^2^ [14], the hydrodynamics of dense crowds have been extensively studied and verified by numerous researchers [9, 23, 24]. In accordance with this, this study introduced a novel hydrodynamic analysis framework that leverages the mixed-type continuum model [25]. This framework was specifically designed to quantitatively assess the hazardous conditions of the crowd during the Seoul Halloween crowd-crush.

#### Scenario setup

**Fig 3:**
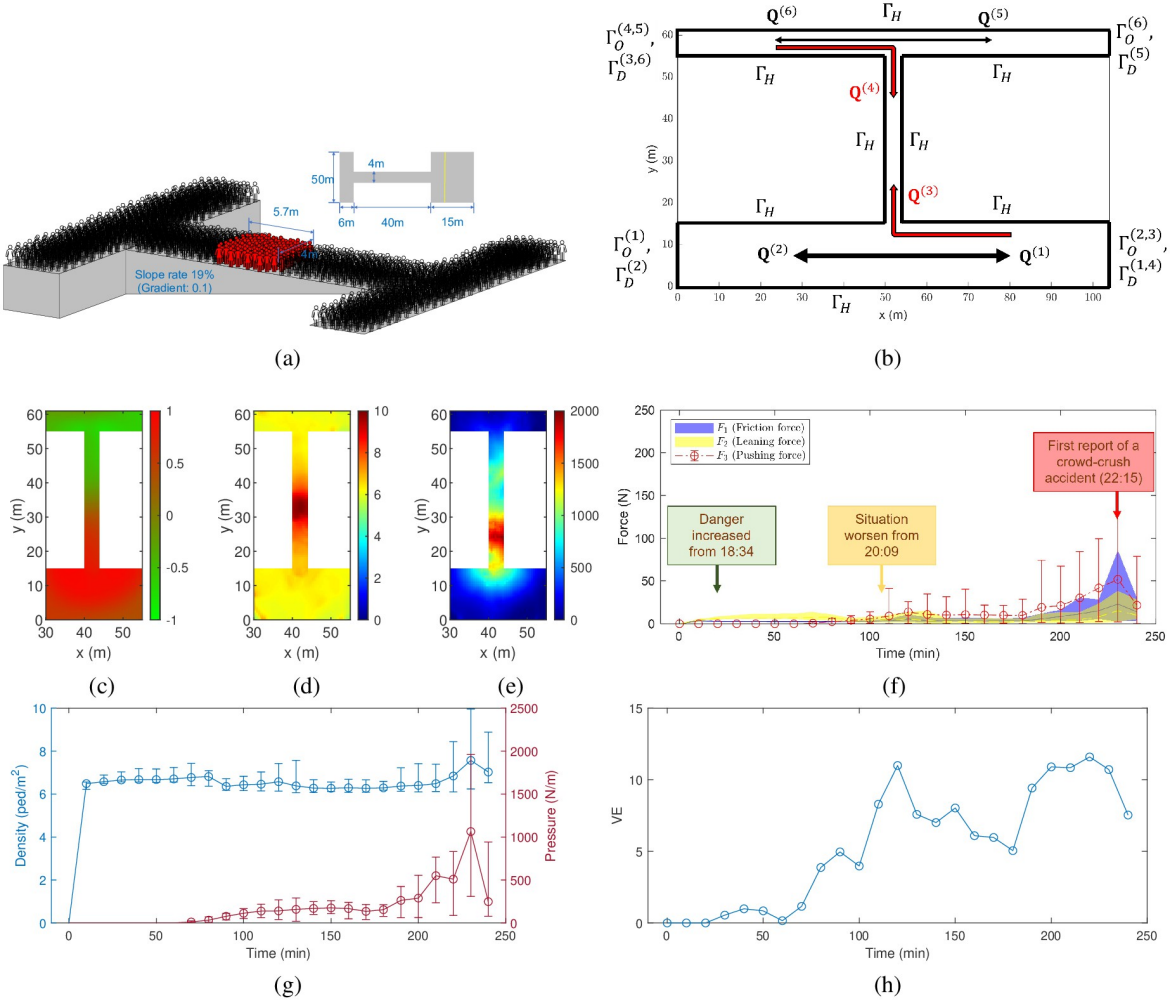
Model and simulation of the Seoul Halloween crowd-crush disaster. (a) Simplified geometry setting for simulation. (b) Directions of the six pedestrian streams, among which the two bidirectional streams in the alley (highlighted in red) are crucial during the reproduction of the crowd crush. (c) Distribution of (*ρ*_*s*_ − *ρ*_*n*_)/(*ρ*_*s*_ + *ρ*_*n*_) at *t* = 230 min, where *ρ*_*n*_ represents the overall density from the street north of the alley and *ρ*_*s*_ represents the overall density from the street south of the alley. (d) Heatmap of overall density in the critical area at *t* = 230 min (unit: ped/m^2^). (e) Heatmap of crowd pressure in the critical area at *t* = 230 min (unit: N/m). (f) Time evolution of the three kinds of forces that were exerted on each pedestrian in the alley (*y* ∈ [15, 55] m). (g) Time evolution of the average density and pressure in the alley. (h) Time evolution of the VE in the alley.

According to the official report [17], over 300 victims were concentrated in an 18.24 m^2^ area of the alley, as shown in Fig 3(a). The red-colored figures indicate the approximate location of the crowd crush, situated in the middle of the alley. Based on this empirical information, the numerical simulation was conducted over a 104 × 61 m^2^ ‘**H**’-shaped area. To ensure detailed spatial resolution and accuracy in the solution of the hydrodynamic model, the domain was discretized into a 208 × 122 grid, utilizing a mesh size of *h* = 0.5 m.

#### Traffic assignment

During the crowd crush, six distinct pedestrian groups with different origins and destinations (OD) were identified, represented by **Q**^(*k*)^, *k* = 1, 2, …, 6. The multidimensional pedestrian flow included two opposing streams on the south street, two on the north street, and two within the alley. As shown in Fig 3(b), the collision of the third and fourth pedestrian streams in the alley is expected to be the most critical juncture in the simulation. Estimating the inflow magnitude at the origin for each pedestrian group during the crowd crush presents a significant challenge due to the scarcity of traffic data [26]. Underestimating the inflow can compromise the reliability of simulation outcomes, while overestimating it may cause congestion to propagate upstream, exceeding the network’s capacity limits. This study utilizes dynamic traffic assignment to adapt to fluctuating traffic conditions near the origin over time. If the density before the origin escalates to congestion levels, the inflow rate is adjusted downward to prevent bottlenecks at the entry point. Conversely, if congestion thresholds are not met, the inflow rate is incrementally increased, potentially inducing congestion within the simulation zone. This approach to dynamic OD assignment avoids the need for actual OD data, providing an effective method for assessing the network’s overall capacity.

#### Boundary conditions

Alongside the inflow boundary conditions at $\Gamma_{O}^{\left(k\right)}$ established by the traffic assignment, the simulation also requires mathematical definitions for conditions at the outflow boundaries $\Gamma_{D}^{\left(k\right)}$, and solid boundaries $\Gamma_{H}^{\left(k\right)}$, as illustrated in Fig 3(b). Referring to the mixed-type continuum model [25], these boundary conditions are associated with route selections based on a cost potential field. At outflow boundaries $\Gamma_{D}^{\left(k\right)}$, the cost potential is set as zero, while at solid boundaries $\Gamma_{H}^{\left(k\right)}$, the cost potential is assigned with a sufficiently high value. This configuration enables the determination of expected walking directions during the dynamic simulation, guided by a reactive dynamic user-equilibrium principle [27].

#### Simulation

The dynamic movement of each pedestrian stream is simulated using a hydrodynamic model framework, as detailed in the **Methods** section. Two crucial aspects of crowd movement are explicitly considered in this model: route strategy and crowd pressure. First, the ground friction resulting from route strategy is represented by adapting the actual motion to the expected speed, which is determined based on the instantaneous density distribution through a reactive user-optimal model [27]. Second, crowd movement is influenced by the gradient of crowd pressure [9]. Notably, the mixed-type continuum model [25] also takes into account the impact of the terrain’s slope within the accident area by integrating a leaning force. The framework establishes a set of partial differential equations (PDEs), as shown in Eq (1). By applying appropriate boundary conditions, these PDEs can be numerically solved using efficient algorithms detailed in S1 Appendix.

$$\{\begin{array}{l} \rho_{t}^{\left(k\right)}+{\left(\rho^{\left(k\right)}u^{\left(k\right)}\right)}_{x}+{\left(\rho^{\left(k\right)}v^{\left(k\right)}\right)}_{y}=0\\ {\left(\rho^{\left(k\right)}u^{\left(k\right)}\right)}_{t}+{\left(\rho^{\left(k\right)}{u^{\left(k\right)}}^{2}+P_{1}^{\left(k\right)}\right)}_{x}+{\left(\rho^{\left(k\right)}u^{\left(k\right)}v^{\left(k\right)}\right)}_{y}=(S_{L}^{(k),1}+S_{R}^{(k),1}+S_{P}^{(k),1})/\bar{m}\\ {\left(\rho^{\left(k\right)}u^{\left(k\right)}\right)}_{t}+{\left(\rho^{\left(k\right)}u^{\left(k\right)}v^{\left(k\right)}\right)}_{x}+{\left(\rho^{\left(k\right)}{v^{\left(k\right)}}^{2}+P_{1}^{\left(k\right)}\right)}_{y}=(S_{L}^{(k),2}+S_{R}^{(k),2}+S_{P}^{(k),2})/\bar{m} \end{array}$$ (1)

The PDEs in Eq (1) describes the temporal dynamics of the crowd states over *t* within a two-dimensional framework, characterized by *x*- and *y*- dimensions. Here, *ρ*^(*k*)^(*x*, *y*, *t*) represents the density of the *k*-th pedestrian stream at a space-time point (*x*, *y*, *t*), while *u*^(*k*)^(*x*, *y*, *t*) and *v*^(*k*)^(*x*, *y*, *t*) denote the velocities of this stream along the *x*- and *y*- dimensions, respectively. The term $P_{1}^{\left(k\right)}$ refers to the pseudo traffic pressure, which is a function of the density. On the right-hand side of the equation, the vectors $S_{L/R/P}^{\left(k\right)}=\left(S_{L/R/P}^{(k),1},S_{L/R/P}^{(k),2}\right)$ correspond to the forces of leaning, ground friction, and the gradient of crowd pressure, respectively. The parameter $\bar{m}$ indicates the average mass of the pedestrian flow. The notation (…)_*t*_, (…)_*x*_, (…)_*y*_, signifies the partial derivatives of the enclosed variables with respect to time and space. It is important to note that the modeling and computation of these forces depend on the instantaneous crowd states. As a result, the dynamics of the crowd can be updated by using numerical algorithms specifically designed for solving Euler equations.

In the static analysis, the model-based reproduction provided a critical understanding of the density and pressure experienced within the alley during the crush event. As illustrated in Fig 3(c), the collision in the alley was apparent through the simulation of multi-directional pedestrian flows. The average density in the alley, around the time of the crowd crush, was calculated to be 7.57 ped/m^2^, peaking at 9.95 ped/m^2^ (Fig 3(d)). Similarly, the average pressure was 1063 N/m, with the maximum pressure reaching 1961 N/m (Fig 3(e)). According to a previous survey [19], a prolonged pressure of 1, 000 N/m or an instantaneous pressure of 2, 500 N/m could cause significant discomfort. Within the crush region, spanning approximately 10 m, the simulation reproduced these high-pressure levels, which were conveyed through density waves. The persistence of this condition over an extended period ultimately led to the crowd-crush disaster in the alley.

In the dynamic analysis, the escalating danger was evident not only through the increasing estimated forces exerted on individual pedestrians (Fig 3(f)), but also by the rise in macroscopic indicators (Fig 3(g) and 3(h)). At the beginning of the simulation (18:34), congestion quickly formed in the alley, with the density reaching over 7 ped/m^2^. However, the situation remained manageable at this stage as panic among the crowd was minimal. As congestion persisted, a sense of panic gradually escalated, acting as a significant external factor contributing to the increase in pressure and turbulence within the hydrodynamic model. By 20:09, some pedestrians were subjected to crowd pressures of approximately 300 N/m, and the VE—a measure of the degree of movement chaos [28]—rose above 10. Simultaneously, the density remained high, leading to a rapid escalation in pressure and turbulence.

By 22:15, the simulation predicted a peak density of 9.95 ped/m^2^ in the crush region, with the pressure in the lower half of this region hitting 1961 N/m. This high level of crowd density reflects the real-world observations from past tragedies, such as the 2010 Love Parade disaster, where peak densities near critical spots were reported around 10 ped/m^2^ [5], and instances of suffocation were associated with pressures exceeding 1000 N/m [19]. Similarly, during the Seoul Halloween incident, fatalities primarily occurred in the lower half of the congested area, a section characterized by the collision of opposing pedestrian streams [29].

In this extremely hazardous scenario, a combined effect of oscillating crowd pressure (2 ∼ 215 N) and leaning force (2 ∼ 39 N) could have potentially triggered a fall. As expected, there was an increase in friction force (5 ∼ 65 N), indicating pedestrians’ efforts to counteract crowd forces. On the individual pedestrian level, intolerable crowd forces could have potentially led to involuntary movement or a fall. At the macroscopic level, the crowd was observed to move chaotically, resulting in the instability or turbulence often seen in such situations [15]. This form of chaos was quantitatively expressed by the VE, the value of which escalated to 10.99 during the simulation.

### Implementation of crowd management strategy

**Fig 4:**
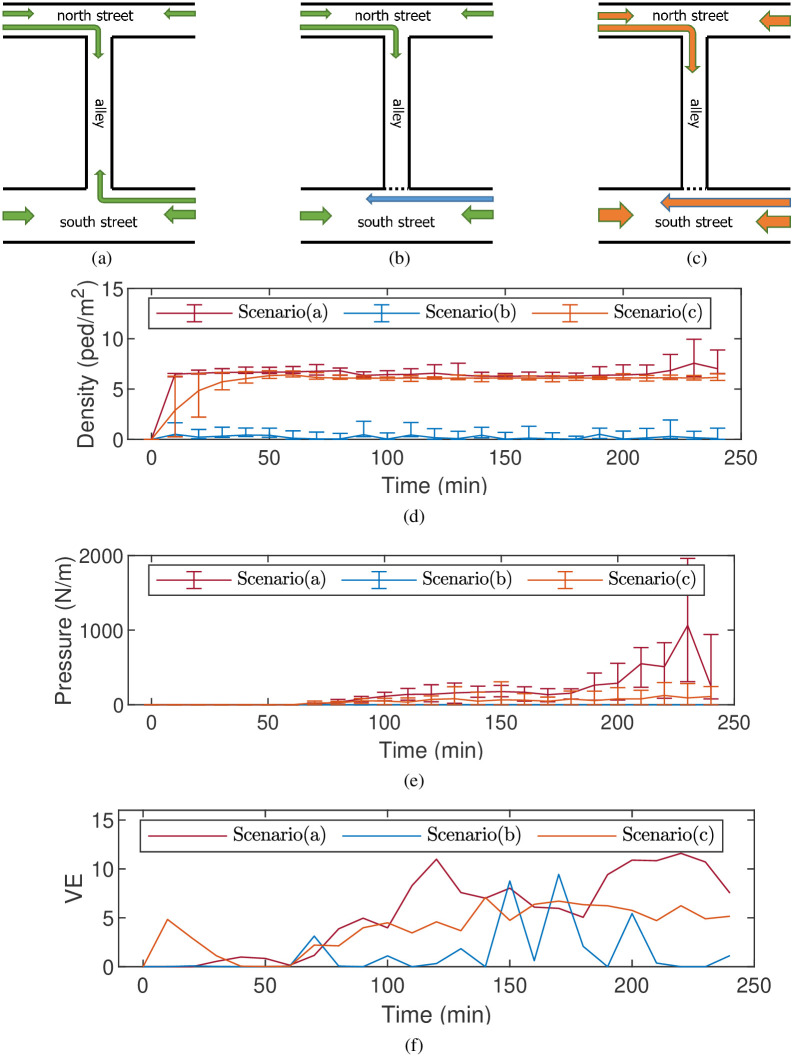
Time evolution of several indicators of dangerous level in the three scenarios. (a) The original scenario. (b) Crowd management strategy assigned. (c) Increasing pedestrian flow in the geometry based on scenario (b). (d) The average overall density in the alley. (e) The averaged crowd pressure *P*_2_ in the alley. (f) The VE [28] in the alley.

The demand for implementing appropriate crowd management strategies is growing, particularly in light of potential crowd disasters. Numerous studies and reports have emphasized the need for preemptive measures to prevent critical situations during such disasters [4, 11]. The empirical report and simulation results from the Seoul Halloween crowd-crush disaster further underscored this need. A detailed analysis of the Seoul disaster revealed that the crush, triggered by the bidirectional pedestrian stream, was a primary cause of crowd risk in the narrow alley. This finding was significant as it provided clear guidance for developing crowd management strategies. As a result, a specific crowd management strategy was designed and tested in controlled experiments. This strategy involved redirecting the pedestrian flow moving towards the south street to the right exit on the north street, as shown in Fig 4(b). The aim of this redirection was to prevent a crush in the alley, which was identified as a major risk factor. In implementing this strategy, it was important to consider the OD estimation. In this configuration, the OD estimation remained the same as in the original scenario (Fig 4(a)). However, the strategy’s implementation inevitably altered the OD estimation. Acknowledging this, an additional scenario (Fig 4(c)) was also considered. This scenario introduced a new dynamic OD estimation designed to generate congestion in the alley, thereby testing the robustness of the crowd management strategy under different conditions.

In Scenario (b), the implemented crowd management strategy led to a significant reduction in congestion. The peak density in the alley throughout the simulation was just 2.86 ped/m^2^, rendering congestion nearly negligible (Fig 4(d)). Meanwhile, the location of this minor congestion shifted to the intersection point between the alley and the south street, a stark contrast to the bidirectional collision region observed in Scenario (a). The congestion at this intersection point, which lacked many solid boundaries, could be quickly alleviated by effectively controlling pedestrian flow on the south street. This swift response and control prevented the formation of a dense crowd, ensuring that no aggregated pressure could develop (Fig 4(e)). Although the VE was highly volatile (Fig 4(f)), it did not indicate a hazardous crowd condition. Instead of signaling potential risk, the instability merely reflected the heterogeneous crowd movement under low-density circumstances.

In Scenario (c), the crowd management strategy was further tested under a new dynamic OD estimation. This estimation was designed to continuously increase the inflow rate until a specified congestion level was reached, potentially leading to congestion within the simulated area. The results, as shown in Table 1, revealed a substantial increase in the average inflow rate, ranging from 125% to 162%. According to police call records [17], panic sentiment began escalating from 8:00 p.m., resulting in a significant reduction in the total inflow in Scenario (a). However, this decrease was barely noticeable in Scenario (c), further emphasizing the effectiveness of the crowd management strategy, even under conditions of heightened panic.

**Table 1:** Comparison of OD estimation in Scenario (a) and Scenario (c). The dynamic OD estimation is implemented in both scenarios to evaluate the network capacity. A considerable increase in the total inflow rate is demonstrated by PCT (percentage).

Time (p.m.)	Scenario (a) (× 100 ped/m/hour)						Scenario (c) (× 100 ped/m/hour)						PCT
	1	2	3	4	5	6	1	2	3	4	5	6	
6:00–7:00	8.1	6.3	3.5	4.7	6.0	8.7	12.2	11.4	0.0	6.0	6.0	10.8	125%
7:00–8:00	6.2	3.9	1.8	3.5	3.9	6.6	9.5	8.0	0.0	4.4	4.1	7.4	129%
8:00–9:00	6.1	4.5	2.6	3.7	2.9	5.9	9.6	7.5	0.0	7.4	4.2	8.0	143%
9:00–10:00	5.2	3.8	3.1	4.6	2.5	5.2	10.4	7.2	0.0	8.8	4.7	8.5	162%

Despite the increased capacity, a decrease in crowd risk was observed, as indicated by key metrics. Congestion in the alley evolved more slowly from *t* = 0 min to *t* = 50 min, resulting in a similar crowd status as in Scenario (a), as depicted in Fig 4(d). Moreover, from *t* = 200 min onwards, when panic sentiment was expected to impact flow patterns, the density did not exhibit any signs of escalation. In addition, by successfully preventing bidirectional crush, the peak pressure (499 N/m) in the alley remained significantly lower than that observed in Scenario (a). At the same time, the evolution of VE suggested that the chaos in the movement was reduced (Fig 4(f)) under this strategy.

By effectively mitigating bidirectional collisions, the implemented crowd management strategy was able to accommodate a higher inflow rate while simultaneously reducing the magnitudes of crowd risk indicators. This improvement in both capacity and safety, even amidst escalating panic sentiment, showcased the potential of this strategy as an effective measure to prevent the Seoul Halloween crowd-crush.

## Methods

**Fig 5:**
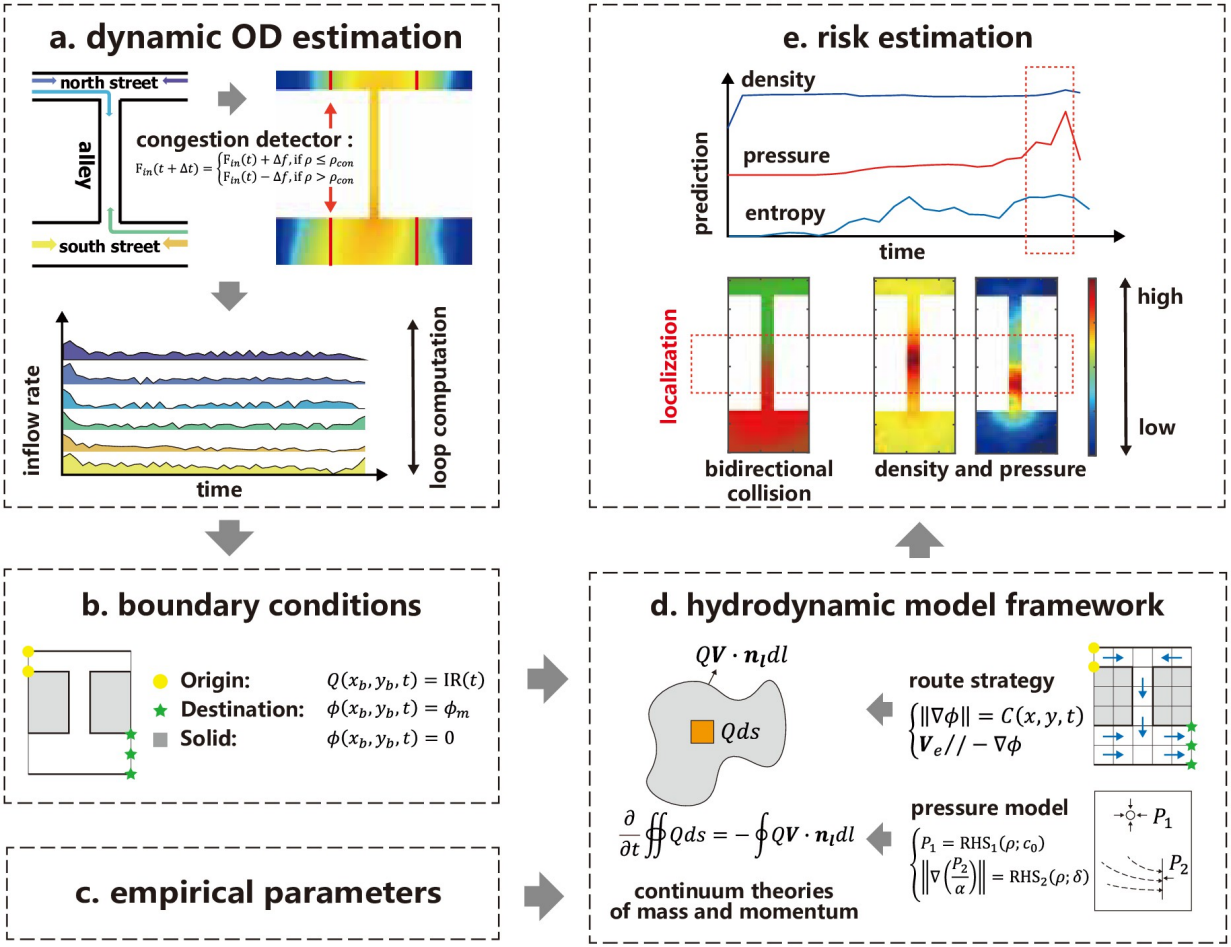
Representation of crowd state prediction and estimation. (a) The dynamic OD estimation provides an inflow rate for each pedestrian stream, determined through the introduction of a congestion detector. (b) The boundary conditions include the OD information and solid boundaries. (c) The model parameters are derived from an extensive review of empirical studies. (d) Utilizing the input data, a hydrodynamic model is employed that explicitly takes into account the route strategy and aggregated pressure. (e) The dynamic evolution of key crowd states and indicators facilitates the identification of potential crowd risks.

As illustrated in Fig 5, this study proposed a hydrodynamic model specifically tailored for multidirectional pedestrian flow. The input data consisted of boundary conditions, such as the dynamic OD estimation for each pedestrian stream, and model parameters, including those associated with the multidirectional pedestrian fundamental diagram. Conversely, the output revealed the evolution of critical crowd states, allowing for the prediction and identification of crowd risk. Drawing on the empirical data from the Seoul Halloween crowd-crush disaster, the model established a series of partial differential equations (PDEs). These equations were solved using numerical algorithms, which enabled the replication of hazardous crowd conditions and the evaluation of crowd management strategies.

### A hydrodynamic model framework

Empirical studies have shown that crowd forces have a dominant effect on crowd movement under high-density conditions [5, 30, 31], so this simulation model investigates the three different kinds of crowd forces separately, namely leaning force **S**_*L*_, ground friction **S**_*R*_ and gradient forces from crowd pressure **S**_*P*_. In general, a crowd can be regarded as several compressible and nonviscous fluids influenced by the abovementioned forces. Consequently, to devise a mathematical description, crowd movement is regarded as following the conservation laws of mass and momentum (Eq (1)). For convenience, let [*e*_1_, *e*_2_, …]^(*k*)^ denote $\left[e_{1}^{\left(k\right)},e_{2}^{\left(k\right)},\ldots \right]$, so Eq (1) can be rewritten as follows:

$$\begin{array}{c} Q_{t}^{\left(k\right)}+F_{x}^{\left(k\right)}+G_{y}^{\left(k\right)}=S^{\left(k\right)}/\bar{m}, \end{array}$$ (2)

where $Q_{t}^{\left(k\right)}=\partial \left({\left[\rho ;\rho u;\rho v\right]}^{\left(k\right)}\right)/\partial t$, indicates the change rate of mass and momentum; $F_{x}^{\left(k\right)}=\partial \left({\left[\rho u;\rho u^{2}+P_{1};\rho uv\right]}^{\left(k\right)}\right)/\partial x$ and $G_{y}^{\left(k\right)}=\partial \left({\left[\rho v,\rho uv,\rho v^{2}+P_{1}\right]}^{\left(k\right)}\right)/\partial y$ indicate the gradients of flow vectors in the *x*- and *y*- dimensions respectively; $\bar{m}$ indicates the average mass of a single pedestrian, which is assumed to be a constant in this study. In the following, detailed assumptions are given for the terms of crowd forces.

$P_{1}^{\left(k\right)}$ -traffic pressure. This is a pseudo-pressure [9, 32] that describes the response of pedestrians to the variations in density around the *k*-th pedestrian group. This relationship is detailed as follows:

$$\begin{array}{c} \sqrt{\frac{dP_{1}^{\left(k\right)}}{d\rho^{\left(k\right)}}}≔c=\{\begin{array}{cc} c_{0}, & \rho^{\left(k\right)}\leq \rho_{0}\\ c_{0}/2, & \rho_{0}<\rho^{\left(k\right)}\leq \rho_{1}\\ 0, & \rho^{\left(k\right)}>\rho_{1} \end{array}, \end{array}$$ (3)

where the parameter *c*_0_ is termed the “sonic speed” which correlates with the maximum speed that waves can propagate through pedestrian flow, as detailed in [33]. The parameters *ρ*_0_ and *ρ*_1_ represent the critical density for physical contact and for crowd turbulence, respectively. The introduction of this term meets the requirement of the continuity assumption and enables the model to describe phase transitions in pedestrian flow dynamics between stable states, such as laminar pedestrian flow, and unstable states [16].

$S_{R}^{\left(k\right)}$ -ground friction generated from route strategy. This describes the tendency [32] of pedestrians to adjust their current walking speed [*u*, *v*]^(*k*)^ to the equilibrium walking speed [*u*_*e*_, *v*_*e*_]^(*k*)^, as formed in Eq (4), where the parameter *τ*^(*k*)^, referred to as the relaxation time [32], governs the intensity of the frictional force. Moving pedestrians are analogized to fluids in a potential field generated by the reactive user-equilibrium route strategy [27], which is given in Eq (5), where *φ*^(*k*)^ is the potential, *f*^(*k*)^(**Q**) is the macroscopic speed-density relationship [34], *g*(*ρ*) is the uncomfort cost [35] related to high density.

$$\begin{array}{c} S_{R}^{\left(k\right)}={\left[0;S_{R}^{1};S_{R}^{2}\right]}^{\left(k\right)}={\left[0;\bar{m}\frac{\rho u_{e}-\rho u}{\tau };\bar{m}\frac{\rho v_{e}-\rho v}{\tau }\right]}^{\left(k\right)} \end{array}$$ (4)

$$\{\begin{array}{l} \left\|\nabla \phi^{\left(k\right)}\|=1/f^{\left(k\right)}(Q)+g(\rho \right)\\ {\left[u_{e},v_{e}\right]}^{\left(k\right)}=f^{\left(k\right)}(Q)\frac{\nabla \phi^{\left(k\right)}}{\left\|\nabla \phi^{\left(k\right)}\right\|} \end{array}$$ (5)

$S_{L}^{\left(k\right)}$ -leaning force. The gradient force caused by a slope could worsen a panic situation. In the Seoul Halloween crowd-crush disaster, it was apparent that pedestrians fell back down the sloping alley [36]. Although little experimentation has been done to quantify the magnitude of the effect of this occurrence, this model assumes that the effect increases after the crowd reaches the critical density for physical contact and is inversely correlated with the relaxation time, as in Eq (6). This term adds some negative value to the velocity along the *y* − dimension in the alley, i.e. *v*_*e*_, at a high-density level.

$$\begin{array}{c} S_{L}^{\left(k\right)}={\left[0,0,\frac{\gamma }{\tau }\bar{m}\rho \right]}^{\left(k\right)}, \end{array}$$ (6)

where *γ*^(*k*)^ = max(0, −0.15(*ρ*^(*k*)^ − *ρ*_0_)/(*ρ*_*m*_ − *ρ*_0_) is the amplification coefficient, *ρ*_0_ and *ρ*_*m*_ are given parameters that denote the critical density for physical contact and the maximum density, respectively.

$S_{P}^{\left(k\right)}$ -gradient of aggregated crowd pressure, as in Eq (7). In dense crowd situations, physical pressure usually plays a dominant role in crowd movement, and extremely high pressure is caused by the aggregation of crowd forces through force chains during crowd disasters [16]. A pressure model was devised for unidirectional cases in [9], the pressure model is proposed for unidirectional cases. The current study further develops the model, as in Eq (8) to describe the aggregation and relaxation properties in multidirectional cases. The given parameters, panic sentiment *δ*^(*k*)^(*x*, *y*, *t*) and the pushing capacity *k*(*ρ*), determine local pushing forces that can propagate through a crowd.

$$\begin{array}{c} S_{P}^{\left(k\right)}={\left[0;S_{P}^{1};S_{P}^{2}\right]}^{\left(k\right)}=\left[0;\frac{\partial P_{2}}{\partial x}\frac{\rho^{\left(k\right)}}{\rho };\frac{\partial P_{2}}{\partial y}\frac{\rho^{\left(k\right)}}{\rho }\right] \end{array}$$ (7)

$$\begin{array}{c} \|\nabla (\frac{P_{2}}{\alpha })\|=\frac{\underset{k}{\text{max}}\;\left(\delta^{\left(k\right)}\right)\cdot k\left(\rho \right)}{\alpha }\cdot \frac{\|\sum_{k}\rho^{\left(k\right)}\nu_{e}^{\left(k\right)}\|}{\rho };\;P_{2}=0\;\text{if}\;\alpha =0 \end{array}$$ (8)

where *α* is the relaxation factor, as determined in Eq (9), and panic sentiment *δ*^(*k*)^(*x*, *y*, *t*) and the pushing capacity *k*(*ρ*) are key influencing factors that determine the magnitude of crowd pressure.

$$\begin{array}{c} \alpha =\{\begin{array}{cc} 1, & \nabla \rho \cdot (\sum_{k}\;\rho^{\left(k\right)}\nu_{e}^{\left(k\right)})\geq 0\\ \text{max}(\frac{\rho -\rho_{0}}{\rho_{m}-\rho_{0}},0), & \nabla \rho \cdot (\sum_{k}\;\rho^{\left(k\right)}\nu_{e}^{\left(k\right)})<0 \end{array} \end{array}$$ (9)

### OD estimation, boundary conditions, and empirical parameters

Based on empirical analysis, the numerical simulation is performed over a 104 × 61 m^2^ ‘**H**’-shaped area, in which there are six pedestrian streams: two opposing streams in the south street, two in the north street, and two in the alley (Fig 3(b)). They were assigned different boundary conditions, i.e., for inflow boundaries $\Gamma_{O}^{\left(k\right)}$, outflow boundaries $\Gamma_{D}^{\left(k\right)}$, and solid boundaries Γ_*H*_.

This study introduces “congestion detectors” situated prior to the origins, which supply information for inflow assignment. As delineated in Eq (10), if the density before the origin reaches the congestion level *ρ*_*con*_, the inflow rate $F_{in}^{\left(k\right)}$ of mass and momentum, which is a vector dependent on *ρ*_*in*_, will begin to diminish to prevent bottlenecks at the origin. Conversely, if the congestion level is not reached, the inflow rate continues to increase, leading to congestion in the simulated area. This dynamic OD estimation method does not necessitate any real-world OD data and can provide an overall capacity estimation of the network. The remaining boundary conditions, encompassing the outflow and solid boundary conditions, align with the precedents set by earlier higher-order continuum models, as referenced in [9].

$$\begin{array}{c} F_{in}^{\left(k\right)}\left(\rho_{in}\left(t+\Delta t\right)\right)=\{\begin{array}{c} F_{in}^{\left(k\right)}\left(\rho_{in}\left(t\right)+\Delta \rho \right),\;\text{if}\;\rho \leq \rho_{con}\\ F_{in}^{\left(k\right)}\left(\rho_{in}\left(t\right)-\Delta \rho \right),\;\text{if}\;\rho >\rho_{con} \end{array} \end{array}$$ (10)

In addition to boundary conditions, the model incorporates parameters and functions, each having unique physical interpretations, and assigns them empirical values. Therefore, the model can reproduce the realistic, dangerous crowd dynamics of the Seoul Halloween crowd-crush disaster. That is, the predicted magnitudes of indicators, such as density, crowd forces, and VE, may not be accurate but nevertheless quantitatively describe the increases in the level of danger in the crowd. The detailed values and empirical evidence are presented below.

- The sonic speed, which is denoted as *c*_0_, is related to the maximum speed that waves can propagate through pedestrian flow [33]. A higher sonic speed leads to higher instability in a crowd, according to a linear stability analysis [9]. As no experiments have been performed on the calibration of sonic speed, this study uses 0.6 m/s to maintain stability at low-density levels.
- The average weight of pedestrians, which is denoted as $\bar{m}$, is set as 65 kg according to the average weight of residents in Seoul [37].
- The critical value of density that allows contact force to arise, which is denoted as *ρ*_0_, adopts 6 ped/m^2^. According to experiments [31, 38], the compression in a crowd starts at approximately 5 ∼ 8 ped/m^2^.
- The critical value of density that allows crowd turbulence to arise, which is denoted as *ρ*_1_, adopts 8 ped/m^2^. According to empirical observations [14, 16], crowd turbulence is observed at approximately 7 ∼ 12 ped/m^2^.
- The maximum density, which is denoted as *ρ*_*m*_, adopts 10 ped/m^2^. During crowd disasters [5, 14, 16], the maximum density observed is approximately 10 ∼ 14 ped/m^2^.
- The function of pushing force related to density is given by Eq (11). Experiments have shown that the pushing capacity of an individual is approximately 30%∼75% of the individual’s weight [14]. This study assumes a range of 200 ∼ 400 N for 6 ∼ 10 ped/m^2^, which is lower than that above, as during crowd disasters it is not likely that all of the pedestrians in a unit area push at once, but the propagation of pushing forces through “force chains” [16] generates a highly dangerous level of crowd pressure.
  $$\begin{array}{c} p\left(\rho \right)=200\sqrt{\text{max}(0,\rho -\rho_{0})} \end{array}$$ (11)
- The function of the fundamental diagram for multidirectional pedestrian flow is given by Eq (12), where $\gamma_{1}^{\left(k\right)}\left(\delta \right)=-0.075\left(1-\delta^{\left(k\right)}\right)-0.045\delta^{\left(k\right)}$. In calm situations, i.e. *δ*^(*k*)^ = 0, the parameters in the model are consistent with those of the model in [34], which was calibrated through experiments. However, in panic situations, the desired speed quickly increases to over twice the normal speed [12], so a second peak is observed in the macroscopic fundamental diagram [16]. Because of the lack of empirical data, this study assumes $\gamma_{1}^{\left(k\right)}=-0.045$ under full panic conditions, which results in a larger desired velocity compared to calm situations.
  $$\begin{array}{c} f^{\left(k\right)}\left(Q\right)=1.034\;\text{exp}\;\left(-\gamma_{1}^{\left(k\right)}\rho^{2}\right)\times \prod_{i=1}^{n}\;\text{exp}\;[-0.019\left(1-\text{cos}\;\varphi_{ik}\right){\left(\rho^{\left(i\right)}\right)}^{2}] \end{array}$$ (12)
- The function of uncomfort cost related to high density is given by Eq (13). The influence of a discomfort cost is considered to be minor during crowd disasters. This study applies the same formulation that has been used by others [9, 32].
  $$\begin{array}{c} g\left(\rho \right)=0.02\rho^{2} \end{array}$$ (13)
- The function of relaxation time related to the panic sentiment is given by Eq (14). In the original Payne–Whitham model, relaxation time is used to characterize driver responses as ranging from approximately 0.51 ∼ 0.89 s, according to experimental calibrations [39, 40]. In reality, relaxation time is considered to be larger because pedestrians are likely to be more relaxed than in experimental environments, as a higher variance is observed in real-world vehicular data than in experimental data [41]. Therefore, this study adopts 5 s in calm situations and 0.5 s in full-panic situations.
  $$\begin{array}{c} \tau^{\left(k\right)}\left(\delta^{\left(k\right)}\right)=0.5\delta^{\left(k\right)}+5\left(1-\delta^{\left(k\right)}\right) \end{array}$$ (14)

Apart from the parameters and functions discussed above, the key exogenous variable, panic sentiment, was defined as in Eq (15) for the third and fourth pedestrian streams, the members of which are traveling on the alley. The unit of time here is minute.

$$\delta^{\left(3,4\right)}(x,y,t)=\{\begin{array}{ll} 0 & t\leq 60\\ 0.7\times (t-60)/60 & 60<t\leq 120\\ 0.7 & 120<t\leq 180\\ 0.7+0.3\times (t-180)/60 & 180<t\leq 240 \end{array}$$ (15)

The hydrodynamic model is ultimately constructed as a series of PDEs, each furnished with appropriate parameters, as well as initial and boundary conditions. These conditions are specified for each pedestrian stream, encompassing inflow boundaries, outflow boundaries, and solid boundaries. Traditional numerical algorithms can be applied to solve the PDE set. The specific numerical algorithms employed to solve these PDEs are detailed in S1 Appendix. The summarized inputs and outputs of the model are presented in Table 2.

**Table 2:** Summary of the inputs and outputs of the model.

Category	Data kind	Data source	Notations
Input	Inflow boundary	Dynamic modeling of OD assignment	$\Gamma_{O}^{\left(k\right)}$ —Location of the origin of the *k*-th pedestrian stream; *f*_*i*_*n*^(*k*)^(*t*)—Inflow rate of the the *k*-th pedestrian stream.
Input	Outflow boundary	Empirical report	$\Gamma_{D}^{\left(k\right)}$ —Location of the destination of the *k*-th pedestrian stream.
Input	Solid boundary	Empirical report	Γ_*H*_—Location of the solid boundary.
Input	Parameters	Empirical assumptions	*c*_0_—Sonic speed $\bar{m}$—Average weight of a single pedestrian; *ρ*_0_—Critical value of density that allows contact force to arise; *ρ*_1_—Critical value of density that allows crowd turbulence to arise; *ρ*_*m*_—Maximum density.
Input	Functions	Empirical assumptions	*p*(*ρ*)—Function of pushing force related to density; *g*(*ρ*)—Function of uncomfort cost related to high density; *f*^(*k*)^(**Q**)—Function of the fundamental diagram for multidirectional pedestrian flow of the the *k*-th pedestrian stream; *τ*^(*k*)^(*δ*^(*k*)^)—Function of relaxation time related to the panic sentiment of the the *k*-th pedestrian stream; *δ*^(3, 4)^(*x*, *y*, *t*)—Evolution of panic sentiment of the the 3rd and 4th pedestrian stream.
Output	Crowd dynamics	-	*ρ*^(*k*)^(*x*, *y*, *t*)—Evolution of density distribution of the *k*-th pedestrian stream; (*u*^(*k*)^(*x*, *y*, *t*), *v*^(*k*)^(*x*, *y*, *t*))—Evolution of speed of the *k*-th pedestrian stream.
Output	Risk indicators of crowd states	-	*VE*(*t*)—Evolution of Velocity Entropy; *P*_2_(*x*, *y*, *t*)—Evolution of aggregated crowd pressure.

The calculation of VE refers to the definition provided in [28], which quantifies the dispersion of velocity distribution in both magnitude and direction. The VE comprises two components: *VE*(*t*) = *E*_*m*_ × *E*_*d*_, where *E*_*m*_ represents magnitude entropy and *E*_*d*_ represents direction entropy *E*_*d*_, as defined in Eqs (16) and (17), respectively. The velocity magnitude is divided into 10 equal-width bins ranging from 0 to 0.1 m/s, and the speed direction is divided into 36 equal-width bins ranging from 0 to 360∘.

$$\begin{array}{c} E_{m}=-\sum_{i=1}^{n_{1}}p_{v}\left(i\right)\;{\text{log}}_{2}\;p_{v}\left(i\right), \end{array}$$ (16)

where *p*_*v*_(*i*) = *h*_*m*_(*i*)/*N*. *h*_*m*_(*i*) indicates the number of moving particles with the velocity magnitude corresponding to the *i*-th bin. *N* indicates the total number of moving particles and *n*_1_ is the total number of velocity magnitude bins.

$$\begin{array}{c} E_{d}=-\sum_{j=1}^{n_{2}}p_{\theta }\left(j\right)\;{\text{log}}_{2}\;p_{\theta }\left(j\right), \end{array}$$ (17)

where *p*_*θ*_(*j*) = *h*_*θ*_(*j*)/*N*. *h*_*θ*_(*j*) indicates the number of moving particles with the velocity magnitude corresponding to the *j*-th bin and *n*_2_ is the total number of angle bins.

## Discussion

This study presents a comprehensive review of the Seoul Halloween crowd crush, utilizing a diverse array of data sources, such as LTE mobile device data and police call records. Unlike previous post-disaster reports [5, 42, 43], a hydrodynamic model, known for its effectiveness in describing dense crowd dynamics [24], is developed and applied to recreate the incident and assess management strategies. This model explicitly considers the impact of physical crowd forces, allowing for a more realistic depiction of hazardous crowd states by quantifying density, turbulence, and pressure at critical locations.

The significance of data-driven crowd management in preventing overcrowding and panic has been increasingly acknowledged [11]. Since computer vision methodologies are not applicable in this crowd disaster, this study examines the use of LTE mobile device data to quantify the crowd density in the Itaewon neighborhood. This data-based tool has proven effective in revealing the magnitude of crowd flow and providing real-time risk estimation from an overall perspective. Moreover, this study explores specific social media activities, such as police calls, to gain insight into crowd sentiment [13, 44]. A comprehensive analysis of police calls indicates escalating panic during the Seoul Halloween crowd-crush.

Real-time data alone is insufficient for implementing effective crowd management strategies. In this methodology, a model-based approach is introduced to identify risk indicators and predict the evolution of crowd states. The empirical survey presented in this study provides essential inputs for the model-based simulation, including the scenario setup and boundary conditions. The consistency of the simulated results with previous empirical studies is achieved by quantifying crowd density [14, 16], crowd turbulence [28], and crowd pressure [19]. Moreover, the model’s effectiveness in enhancing crowd safety is demonstrated by numerical tests that assess the impact of various crowd management strategies.

However, the study acknowledges limitations in quantitatively predicting precise crowd conditions at critical junctures due to the adoption of simplified assumptions and boundary conditions. Specifically, prediction of the inflow rates, as outlined in Table 1, might differ from the actual inflow, which could lead to inaccuracies in forecasting the onset of hazardous crowd dynamics. Additionally, the inherent unpredictability of pedestrian behavior, influenced by psychological factors, introduces a significant level of stochasticity, complicating the task of achieving quantitative alignment with actual events during the crowd crush. To overcome these challenges and enhance the model’s predictive accuracy and utility, future research should focus on developing a real-time evaluation framework. This framework would employ short-term predictions of crowd dynamics, informed by data-driven insights, to provide a more reliable and interpretable basis for crowd management decision-making.

Finally, the primary causes of the crowd disaster identified in this study are discussed as follows.

- The relaxation of social distancing measures, combined with the festive atmosphere of Halloween, resulted in a substantial increase in local population density. Empirical reports, based on LTE mobile-signal data, indicate a population surge of over 300% in Itaewon-Dong.
- There was a significant delay in crowd reallocation around the alley. Despite frequent calls to the police that alerted them to the panicked situation, as discussed in the empirical report, crowd management strategies were not effectively implemented.
- A bidirectional collision in the alley led to the entrapment and collision of two separate crowd groups. Through hydrodynamic modeling, the reproduction showed a peak density of 9.95 ped/m^2^ and pressure of 1961 N/m at the collision point, as depicted in Fig 3.
- The opening of driveways to pedestrians on the south street resulted in an increased flow rate of people.
- The incident occurred in the evening, a period typically associated with decreased visibility.
- A slight slope intensified the crowd forces on the downstream crowd.

While many of these factors are difficult to modify, this study suggests proactive measures that can be taken to predict and prevent such disasters.

- Monitor population densities through LTE data and identify potential risk locations to implement crowd management strategies.
- Keep the public informed about the situation and issue warnings regarding the state of the crowd.
- Manage the flow of people in the alley. Narrow pathways and bidirectional traffic should be avoided, and if necessary, the alley should be completely closed off.
- Respond promptly to calls to the police, as they can provide real-time information about crowd dynamics.
- Develop more effective methods for communicating with the crowd.

As the COVID-19 pandemic subsides, events attracting large crowds in complex environments are becoming increasingly common. Consequently, it is essential to employ data-driven methods to predict and prevent similar crowd-related disasters in the future.

## Supporting information

S1 Table Supplementary table: An integrated timeline before the Seoul Halloween crowd-crush. This table integrates data from historical emergency calls, the timeline of government bodies before the Seoul Halloween crowd-crush disaster, and the de facto population in Itaewon in October 2022 to describe comprehensive timelines before the disaster.
(PDF)

S1 Appendix Supplementary method: Numerical algorithm for the hydrodynamic model. The hydrodynamic model developed in this study forms a set of PDEs that are solved by the mixed-type finite difference method coupled with the fast sweeping method. Details of the model formulation and solution algorithms are described in the Supplementary Method.
(PDF)
