# Supplementary material for: Unraveling the causes of the Seoul Halloween crowd-crush disaster
Source: PLoS One. 2024 Jul 12;19(7):e0306764. doi: 10.1371/journal.pone.0306764 (PMC11244771; doi:10.1371/journal.pone.0306764)
Supplement: S1 Table — This table integrates data from historical emergency calls, the timeline of government bodies before the Seoul Halloween crowd-crush disaster, and the de facto population in Itaewon in October 2022 to describe comprehensive timelines before the disaster. (PDF) [file pone.0306764.s001.pdf]

S1 Table for
Unraveling the Causes of the Seoul Halloween Crowd-Crush Disaster

Haoyang Liang[1,2], Seunghyeon Lee[3*], Jian Sun[1], S.C. Wong[2*]

[1]Key Laboratory of Road and Traffic Engineering, Ministry of Education, Tongji University, Shanghai, China.
[2*]Department of Civil Engineering, The University of Hong Kong, Hong Kong SAR, China.
[3*]Department of Transportation Engineering, University of Seoul, Seoul, South Korea.

*Corresponding author(s). E-mail(s): seunghyeon.lee@uos.ac.kr; hhecwsc@hku.hk;
Contributing authors: lianghy@connect.hku.hk; sunjian@tongji.edu.cn;

1 S1 Table. Supplementary Table: An integrated timeline before the Seoul Halloween crowd-crush

To better understand the circumstances surrounding the disaster and the response of government agencies and emergency services, data from multiple sources, such as overcrowding-related calls made before and during the disaster, the timeline presented by the government bodies, and the de facto population in Itaewon on the day of the disaster, were used in the investigation to determine the causes and details of the disaster as presented in Table S1.

Table S1: **An integrated timeline before the Seoul Halloween crowd-crush disaster**

No	Time	Sources	Situations
1	00:59	De facto population	The de facto populations are 7,881 and 7,586 in Areas 2 and 3, respectively, which are 2.4 and 3.5 times more than their respectively than the averages in October 2022., in areas 2 and 3, respectively. People have already gathered to celebrate Devil's Night, which is Halloween Eve. [1]
2	08:00	De facto population	The de facto populations have decreased to 1,701 and 1,480 in Areas 2 and 3, respectively, and are now similar to the average in October 2022. [1]
3	15:00	De facto population	The de facto populations have increased to 4,010 and 3,593 in Areas 2 and 3, respectively, which are 1.4 and 1.7 times their respective averages in October 2022. [1]
4	16:00	De facto population	The de facto populations have increased to 5,766 and 4,959 in Areas 2 and 3, respectively, which are 1.9 and 2.2 times their respective averages in October 2022. [1]

Continued on next page

Table S1: **An integrated timeline before the Seoul Halloween crowd-crush disaster** (Continued)

5	17:00	De facto population	The de facto populations have increased to 8,475 and 7,730 in Areas 2 and 3, respectively, which are 2.7 and 3.2 times their respective averages in October 2022 [1]
6	18:00	De facto population	The de facto populations have increased to 11,468 and 11,206 in Areas 2 and 3, respectively which are 3.4 and 4.1 times their respective averages in October 2022. [1]
7	18:34	Overcrowding-related calls	In the first overcrowding-related call, the caller reports that someone might be crushed and that the police need to control the situation. [2] People would have already felt severe pressure in the crowd in Itaewon as the number of people had increased to over four times the average for October 2022.
8	18:37	National Policy Agency	Three minutes after the first call, police officers are dispatched to control the situation. According to the report from the National Police Agency, the tourist police force of 10 members was deployed in Itaewon to control the Halloween crowd. [2]
9	19:00	De facto population	The de facto populations have increased to 14,046 and 13,669 in Areas 2 and 3, respectively, which are 3.9 and 4.7 times their respective averages in October 2022. [1]
10	19:34	News article	One news article reports that requests for the deployment of police traffic control units were ignored two hours before the disaster. However, police traffic control units were deployed in Yongsan district to control the crowd in a candlelight protest march. [3]
11	20:00	De facto population	The de facto populations have increased to 15,066 and 14,995 in Areas 2 and 3, respectively, which are 3.9 and 5.1 times their respective averages in October 2022. [1]
12	20:09	Overcrowding-related calls	In the second overcrowding-related call, the caller reports that people are pushing each other and falling over, and that some are getting hurt. As the de facto populations drastically increased in both areas after 17:00, people were starting to fall over and suffer injury in Itaewon. [2]
13	20:25	Situations in the adjacent area	The candlelight protest march ends in Yongsan district. We presume that the police traffic control units who were controlling the protest march might now have been available to be deployed to Itaewon to control the crowd. [4]
14	20:33	Overcrowding-related calls	In the third overcrowding-related call, the caller reports that the area is densely populated and that people are closely packed together. So far, all of the callers have reported that the area is densely populated and that it is a dangerous situation. [2]
15	20:37	Communications between police and fire departments	The police agency asks for the cooperation of fire departments to handle the crowd in Itaewon but their requests are declined. [5] We suppose that the fire department declined the requests because the callers only asked for personnel to be dispatched to Itaewon to manage the crowd.
16	20:48	Seoul Metropolitan Police Agency	Seoul Metropolitan Police Agency deploys 50 personnel, including criminal and drug crime investigation units, to Yongsan, Dongjak, Gangbuk, and Gwangjin police stations. [6]

Continued on next page

Table S1: **An integrated timeline before the Seoul Halloween crowd-crush disaster** (Continued)

17	20:53	Overcrowding-related calls	According to the fourth overcrowding-related call, people have begun experiencing physical discomfort in an alleyway in Itaewon. [2]
18	21:00	De facto population	The de facto populations have increased to 16,001 and 15,544 in Areas 2 and 3, respectively, which are 4.0 and 5.2 times their respective averages in October 2022. [1]
19	21:00	Overcrowding-related calls	According to the fifth overcrowding-related call, more people are asking for police officers to come to control the situation. [2]
20	21:01	Seoul Metropolitan Police Agency	According to the report from the Seoul Metropolitan Police Agency, police officers focus on preventing people from pouring onto the streets.[7] We suggest that this could have increased the crowd pressure on sidewalks.
21	21:02	Overcrowding-related calls	In the sixth call overcrowding-related call, an hour before the disaster occurs, the caller is the first to express concern about potential fatalities [2] The population density in Itaewon is approximately 5 times the average density for the neighborhood, which could have increased the crowd pressure.
22	21:05	Seoul Metropolitan Police Agency	The head of the Itaewon police station orders police officers to ease congestion at exit 2 of Itaewon Station and confirm the situation after doing so. We suggest that given the de facto population, even if the police officers had started to control the crowd to ease pedestrian congestion, this would not have significantly decreased the size of the crowd. [6]
23	21:07	Overcrowding-related calls	A caller reports that there are too many people, and “I'm in danger of being crushed.” [2] This indicates that people felt they were at risk of being in a crushing accident.
24	21:10	Overcrowding-related calls	A caller reports that the situation at the Halloween festival is serious and that children are getting trampled inside. [2] This indicates that people have seen children who have been crushed in the crowd and that the situation is becoming serious.
25	21:25	Two police officers	The head of the Itaewon police station adds two more police officers to Itaewon Station exit 1. [8] This is 50 minutes before the disaster and we infer that this measure did not help to reduce the crowd pressure on the sidewalk.
26	21:32	Seoul Metropolitan Police Agency	Yongsan police agency calls the Seoul Metro and requests non-stop passage at Itaewon Station. [9] However, this request is declined by the local manager. Accordingly, we assume that people who did not know about the situation in Itaewon continued to gather there to celebrate Halloween.
27	21:34	20 police officers	The Seoul Metropolitan Police Agency deploys 20 military police officers from a traffic control and traffic mobile unit to Itaewon for traffic management. [10] We think that 20 military police officers would not have been effective for controlling the more than 30,000 people who were by now in Itaewon.

Continued on next page

Table S1: **An integrated timeline before the Seoul Halloween crowd-crush disaster** (Continued)

28	21:51	Overcrowding-related calls	A caller reports that there are "too many people here, so I think you need to control the crowd." [2] Other callers kept asking for crowd control in Itaewon, too, which implies that the number of police officers who were controlling the crowd was not sufficient to relieve the crowd pressure at this time.
29	22:00	De facto population	The de facto populations have increased to 14,689 and 16,360 in Areas 2 and 3, respectively, which are 3.8 and 5.5 times their respective averages in October 2022 [1] Moreover, 52 police officers are in Itaewon to control the crowd at this time. [6]
30	22:00	Overcrowding-related calls	A caller reports that a large number of people are pushing and shoving in the narrow alleyway where the disaster occurred. [2] The alleyway connects the Itaewon main street and the exit of Itaewon Station. We demonstrate the dynamic propagation of crowd pressure and the dynamic distribution of the crowd density in the narrow alleyway.
31	22:11	Overcrowding-related calls	A caller reports that "it feels like we're going to be crushed and everyone's going crazy here." [2] This is the last overcrowding-related call, immediately before the disaster.
32	22:15	Time of the stampede incident	This time was officially announced as the time of the stampede incident. [11] Fifty-nine emergency calls were reported around this time with keywords such as "death," "rescue," and "quickly." Our models replicate the collapse and temporal evolution of the crowd in the alleyway with the given data sets.
33	22:18	Ambulance departed	The first ambulance departs but does not arrive until 22:42, due to the high density of people nearby the disaster location [12]
34	23:00	De facto population	The de facto populations have decreased to 9,719 and 12,627 in Areas 2 and 3, respectively, which are 2.7 and 6.5 times their respective averages in October 2022. [1]
35	23:59	Statistics in Seoul Metro	The total numbers of boarding and alighting passengers were over 48,000 and 81,000, respectively, at Itaewon Station on October 29, 2022, which are five and eight times their respective averages in October 2022. [13]

References

[1] SMG. **Seoul Metropolitan Government** de facto population data sets. https://data.seoul.go.kr/dataList/OA-14979/F/1/datasetView.do (2023). Accessed: 2023-03-20.

[2] Hankookilbo. The first emergency call. https://youtu.be/sr6UHmnVlu4 (2023). Accessed: 2023-03-20.

[3] Hankyoreh. Asking for the deployment of the police traffic control unit was ignored two hours before the itaewon disaster. https://www.hani.co.kr/arti/society/society_general/1065626.html (2023). Accessed: 2023-03-20.

[4] Nocutnews. About the candlelight protest march. https://www.nocutnews.co.kr/news/5843053 (2023). Accessed: 2023-03-20.

[5] Hankyoreh. The police agency asked for the cooperation of fire departments to handle the crowd in the itaewon district. https://www.hani.co.kr/arti/area/capital/1066072.html (2023). Accessed: 2023-03-20.

[6] Newstapa. Overall timelines on 29 october 2022 summarized by newstapa. https://pages.newstapa.org/n2211_timeline/ (2023). Accessed: 2023-03-20.

[7] Hankyoreh. The police agency focused on extending the sidewalk to the road. https://www.hani.co.kr/arti/society/society_general/1069450.html (2023). Accessed: 2023-03-20.

[8] JTBC. The police officers were deployed at itaewon station. https://news.jtbc.co.kr/article/article.aspx?news_id=NB12090740 (2023). Accessed: 2023-03-20.

[9] Hankyoreh. Yongsan police agency called the seoul metro and requested a non-stop pass at itaewon station. https://www.hani.co.kr/arti/society/society_general/1070296.html (2023). Accessed: 2023-03-20.

[10] Hankyoreh. The seoul metropolitan police agency deployed 20 military police officers to the itaewon area. https://www.hani.co.kr/arti/society/society_general/1067877.html (2023). Accessed: 2023-03-20.

[11] Hankyoreh. The time of the stampede incident. https://www.hani.co.kr/arti/society/society_general/1065976.html (2023). Accessed: 2023-03-20.

[12] Hankyoreh. The first ambulance departed at 22:18. https://www.hani.co.kr/arti/society/society_general/1065868.html (2023). Accessed: 2023-03-20.

[13] SMG. **Seoul Metropolitan Government** the number of passengers in seoul metro systems. https://data.seoul.go.kr/dataList/OA-12914/S/1/datasetView.do (2023). Accessed: 2023-03-20.
