# Supplementary material for: Unraveling the causes of the Seoul Halloween crowd-crush disaster
Source: PLoS One. 2024 Jul 12;19(7):e0306764. doi: 10.1371/journal.pone.0306764 (PMC11244771; doi:10.1371/journal.pone.0306764)
Supplement: S1 Appendix — The hydrodynamic model developed in this study forms a set of PDEs that are solved by the mixed-type finite difference method coupled with the fast sweeping method. Details of the model formulation and solution algorithms are described in the Supplementary Method. (PDF) [file pone.0306764.s002.pdf]

S2 Appendix for
Unraveling the Causes of the Seoul Halloween Crowd-Crush Disaster

Haoyang Liang[1,2], Seunghyeon Lee[3*], Jian Sun[1], S.C. Wong[2*]

[1]Key Laboratory of Road and Traffic Engineering, Ministry of Education, Tongji University, Shanghai, China.
[2*]Department of Civil Engineering, The University of Hong Kong, Hong Kong SAR, China.
[3*]Department of Transportation Engineering, University of Seoul, Seoul, South Korea.

*Corresponding author(s). E-mail(s): seunghyeon.lee@uos.ac.kr; hhecwsc@hku.hk;
Contributing authors: lianghy@connect.hku.hk; sunjian@tongji.edu.cn;

1 S2 Appendix. Supplementary Method: Numerical algorithm for the hydrodynamic model

1.1 Summary of the hydrodynamic model

The hydrodynamic model developed for the Seoul Halloween crush is formulated as a set of partial differential equations (PDEs) with appropriate initial and boundary conditions. Because the model corresponds to a multidirectional flow system, the PDE set for each pedestrian stream includes the conservation laws of mass and momentum; expected speed with pressure potential; and inflow boundary $\Gamma_O^{(k)}$, outflow boundary $\Gamma_D^{(k)}$ and common solid boundary Γ_H conditions.

1.1.1 Mass and momentum conservation

Equation (S1) presents the conservation laws of mass and momentum for the k-th pedestrian group.

$$\mathbf{Q}_t^{(k)} + \mathbf{F}_x^{(k)} + \mathbf{G}_y^{(k)} = \mathbf{S}^{(k)}/\bar{m}; \quad (\mathbf{F}^{(k)}, \mathbf{G}^{(k)}) = (\mathbf{F}_O^{(k)}, \mathbf{G}_O^{(k)}) \quad \text{if} \quad (x,y) \in \Gamma_O^{(k)} \tag{S1}$$

where

$$\mathbf{Q}^{(k)} := \begin{bmatrix} q_1 \\ q_2 \\ q_3 \end{bmatrix}^{(k)} = \begin{bmatrix} \rho \\ \rho u \\ \rho v \end{bmatrix}^{(k)}, \mathbf{S}^{(k)} := \begin{bmatrix} 0 \\ 0 \\ \bar{m}\frac{\gamma\rho}{\tau} \end{bmatrix}^{(k)} + \begin{bmatrix} 0 \\ \bar{m}\frac{f(\mathbf{Q})q_1\nu_x - q_2}{\tau} \\ \bar{m}\frac{f(\mathbf{Q})q_1\nu_y - q_3}{\tau} \end{bmatrix}^{(k)} - \begin{bmatrix} 0 \\ \frac{\rho^{(k)}}{\rho}\frac{\partial P_2}{\partial x} \\ \frac{\rho^{(k)}}{\rho}\frac{\partial P_2}{\partial y} \end{bmatrix} \tag{S2a}$$

$$\mathbf{F}^{(k)} := \begin{bmatrix} q_2 \\ \frac{q_2^2}{q_1} + h(q_1) \\ \frac{q_2 q_3}{q_1} \end{bmatrix}^{(k)} = \begin{bmatrix} \rho u \\ \rho u^2 + P_1 \\ \rho uv \end{bmatrix}^{(k)}, \mathbf{G}^{(k)} := \begin{bmatrix} q_3 \\ \frac{q_2 q_3}{q_1} \\ \frac{q_2}{q_1} + h(q_1) \end{bmatrix}^{(k)} = \begin{bmatrix} \rho v \\ \rho uv \\ \rho v^2 + P_1 \end{bmatrix}^{(k)} \tag{S2b}$$

, $h(\rho^{(k)}) = \int_0^{\rho^{(k)}} c^2\, dx$ and $(\nu_x, \nu_y)^{(k)} = (u_e, v_e)^{(k)} / \|(u_e, v_e)^{(k)}\|$.

1.1.2 Equilibrium speed considering pressure potential

Two static Eikonal equations are included to take into account the route strategy and aggregated pushing potential.

First, this predictive user-equilibrium model is applied to determine the expected movement direction $\boldsymbol{\nu}_e^{(k)} = (\nu_x, \nu_y)^{(k)}$, as indicated in Equation (S3).

$$\|\nabla\phi_e^{(k)}\| = g(\rho) + 1/f^{(k)}(\mathbf{Q}); \quad \phi_e^{(k)} = 0 \quad \text{if} \quad (x, y) \in \Gamma_D^{(k)} \tag{S3a}$$

$$\boldsymbol{\nu}_e^{(k)} = -\nabla\phi_e^{(k)}/\|\nabla\phi_e^{(k)}\| \tag{S3b}$$

where $g(\rho)$ indicates the local discomfort cost associated with high density.

Second, the crowd pressure for the overall pedestrian flow is determined through Equation (S4).

$$\left\|\nabla\left(\frac{P_2}{\alpha}\right)\right\| = \frac{\max_k(\delta^{(k)}) \cdot p(\rho)}{\alpha} \cdot \frac{\|\sum_k \rho^{(k)}\boldsymbol{\nu}_e^{(k)}\|}{\rho}; \quad P_2 = 0 \quad \text{if} \quad \alpha = 0 \tag{S4}$$

where α is the relaxation factor, defined in Equation (S5), $p(\rho)$ indicates the relationship between pushing capacity and density, and $\delta^{(k)} \in [0, 1]$ describes the panic sentiment.

$$\alpha = \begin{cases} 1, & \nabla\rho \cdot (\sum_k \rho^{(k)}\nu_e^{(k)}) \geq 0 \\ \max(\frac{\rho-\rho_0}{\rho_m-\rho_0}, 0), & \nabla\rho \cdot (\sum_k \rho^{(k)}\nu_e^{(k)}) < 0 \end{cases} \tag{S5}$$

1.2 Solution algorithm: Mixed-type finite difference model

Given the above-described higher-order continuum model, the Seoul Halloween crowd-crush disaster is then formulated as a set of PDEs with appropriate boundary conditions. This model-based simulation introduces several numerical algorithms to solve nonlinear problems numerically.

First, (S1) is discretized as in (S6). The second-order total variation diminishing (TVD) Runge–Kutta scheme, as presented in Algorithm 1, is used for time integration. At each time step, the crowd state including all pedestrian streams $\mathbf{Q}_n$ is updated with $\mathbf{Q}_{n+1}$ until the simulation time reaches its maximum.

$$\begin{aligned} \mathbf{L}^{(k)}(\mathbf{Q}, t) = \frac{d\mathbf{Q}}{dt} &= -(\mathbf{F}_x^{(k)} + \mathbf{G}_y^{(k)}) + \mathbf{S}^{(k)}/\bar{m} \\ &\sim -\frac{1}{h}(\hat{\mathbf{F}}^{(k)}_{i+\frac{1}{2},j} - \hat{\mathbf{F}}^{(k)}_{i-\frac{1}{2},j}) - \frac{1}{h}(\hat{\mathbf{G}}^{(k)}_{i,j+\frac{1}{2}} - \hat{\mathbf{G}}^{(k)}_{i,j-\frac{1}{2}})) + \frac{\mathbf{S}^{(k)}(\mathbf{Q})}{\bar{m}} \end{aligned} \tag{S6}$$

Algorithm 1 Second-order TVD Runge–Kutta Scheme

```
n ← 0; Q_0^(k) ← 0                                   ▷ Initially, the simulation area is empty
t_0 ← 0; Δt_0 ← 0.01
while n = 0; t_n ≤ t_max; n++ do
    while k = 1; k ≤ N; k++ do
        Q̃^(k) ← Δt_n × L^(k)(Q_n, t_n)
    end while
    while k = 1; k ≤ N; k++ do
        Q_{n+1}^(k) ← (Q_n^(k))/2 + (Q̃^(k) + Δt_n × L^(k)(Q̃, t_n + Δt_n))/2
    end while
    Δt_n ← CFL(h/α)                                  ▷ Requirement of the CFL condition
    t_{n+1} ← t_n + Δt_n
end while
```

On the right-hand side of (S6), the Godunov fractional step method is introduced to numerically solve the Eikonal equations ((S3) and (S4)), as in Algorithm 2.

Algorithm 2 Godunov Fast Sweeping Method

```
φ_(n_x×n_y) ← 10^12                                   ▷ Initially, the potential is at a maximum
while NIT = 0; NORM(φ^new − φ^old) ≤ 10^−9; NIT + + do
    while (i, j) in the GS sequences do
        T_x ← min(φ_{i−1,j}, φ_{i+1,j})
        T_y ← min(φ_{i,j−1}, φ_{i,j+1})
        if |T_x − T_y| ≥ C_(i,j) × h then
            φ^new_(i,j) ← min(T_x, T_y) + C_(i,j) × h
        else
            φ^new_{i,j} ← (T_x + T_y + sqrt(2 C^2_{i,j} h^2 − (T_x − T_y)^2))/2
        end if
        φ^new_{i,j} ← 0 if (x_i, y_j) ∈ Γ_D              ▷ Fixed boundary condition during iterations
    end while
end while
calculate ∇φ(x, y) by the central difference method
```

Finally, the mixed-type FDM, which considers the phase transition between hyperbolicity and ellipticity [1], is presented in Algorithm 3 for approximation of the numerical fluxes. In the hyperbolic region, the eigenvalues of the Jacobi matrix are real and unique. The numerical fluxes are approximated via the traditional local Lax–Friedrichs (LLF) scheme in the characteristic space. In the elliptic region, the Jacobi matrix becomes singular. A new splitting scheme is introduced to capture instability in this region.

Algorithm 3 Mixed-type Finite Difference Method

while $i = -\frac{1}{2}; i \leq (n_x + \frac{1}{2}); i++$ **do**
  while $j = 1; j \leq n_y; j++$ **do**
    if $\rho^{(all)}_{i+\frac{1}{2},j} \leq \rho_E$ **then** ▷ Hyperbolic region
      Decompose the characteristics of Jacobi matrix $\mathbf{J_F}(\mathbf{Q}) = \mathbf{R}\boldsymbol{\Lambda}\mathbf{R}^{-1}$
      $\mathbf{T}^{(\mathbf{Q})}_{s,j} \leftarrow \mathbf{R}(\mathbf{Q}^{(k)}_{i+\frac{1}{2},j})\mathbf{Q}^{(k)}_{s,j}; \mathbf{T}^{(\mathbf{F})}_{s,j} \leftarrow \mathbf{R}(\mathbf{Q}^{(k)}_{i+\frac{1}{2},j})\mathbf{F}^{(k)}_{s,j}$ ▷ Characteristic projection
      $\lambda^{k,H}_{i+\frac{1}{2},j} \leftarrow \max_s(\max(\boldsymbol{\Lambda}(\mathbf{Q}^{(k)}_{s,j})))$
      $\hat{\mathbf{T}}_{i+\frac{1}{2},j} \leftarrow \frac{1}{2}(\mathbf{T}^{(\mathbf{F})}_{i,j} + \mathbf{T}^{(\mathbf{F})}_{i+1,j} - \alpha^{k,H}_{i+\frac{1}{2},j}(\mathbf{T}^{(\mathbf{Q})}_{i,j} - \mathbf{T}^{(\mathbf{Q})}_{i+1,j}))$ ▷ LLF scheme
      $\hat{\mathbf{F}}^{(k)}_{i+\frac{1}{2},j} \leftarrow \mathbf{R}(\mathbf{Q}^{(k)}_{i+\frac{1}{2},j})\hat{\mathbf{T}}_{i+\frac{1}{2},j}$
    else ▷ Non-hyperbolic region
      $\boldsymbol{\Lambda}^{k,E} \leftarrow [\lambda_0 + M, \lambda_0, \lambda_0]^T$
      $\hat{\mathbf{F}}^{(k)}_{i+\frac{1}{2},j} \leftarrow \frac{1}{2}(\mathbf{F}_{i,j} + \mathbf{F}_{i+1,j} - \boldsymbol{\Lambda}^{k,E}(\mathbf{Q}_{i,j} - \mathbf{Q}_{i+1,j}))$ ▷ LF for non-hyperbolic region
    end if
  end while
end while
calculate the numerical fluxes $\hat{\mathbf{G}}^{(k)}_{i,j+\frac{1}{2}}$ along the y direction
$\frac{1}{h}(\hat{\mathbf{F}}^{(k)}_{i+\frac{1}{2},j} - \hat{\mathbf{F}}^{(k)}_{i-\frac{1}{2},j}) + \frac{1}{h}(\hat{\mathbf{G}}^{(k)}_{i,j+\frac{1}{2}} - \hat{\mathbf{G}}^{(k)}_{i,j-\frac{1}{2}}) \sim \nabla \cdot (\mathbf{F}^{(k)}, \mathbf{G}^{(k)})$

calculate cost potential $\nabla\phi^{(k)}(x, y)$ and pressure $\nabla P_2(x, y)$ through Algorithm 2
$\mathbf{S}^{(k)} \leftarrow \mathbf{S}_R(\nabla\phi^{(k)}(x, y)) + \mathbf{S}_P(\nabla P_2(x, y)) + \mathbf{S}_L(\mathbf{Q}(x, y))$
$\mathbf{L}^{(k)}(\mathbf{Q}, t) \leftarrow (\mathbf{S}^{(k)}/\bar{m} - \nabla \cdot (\mathbf{F}^{(k)}, \mathbf{G}^{(k)}))$

References

[1] Shu, C. & Adrminimration, S. A numerical method for systems of conservation laws of mixed type admitting hyperbolic flux splitting. *Journal of Computational Physics* **100**, 424–429 (1992).
